# Epigenetic Modifications in Essential Hypertension

**DOI:** 10.3390/ijms17040451

**Published:** 2016-03-25

**Authors:** Ingrid A. Wise, Fadi J. Charchar

**Affiliations:** Faculty of Science & Technology, Federation University Australia, University Drive, Mount Helen, VIC 3350, Australia; i.wise@federation.edu.au

**Keywords:** essential hypertension, epigenetics, DNA methylation, histone modifications, non-coding RNA, microRNAs

## Abstract

Essential hypertension (EH) is a complex, polygenic condition with no single causative agent. Despite advances in our understanding of the pathophysiology of EH, hypertension remains one of the world’s leading public health problems. Furthermore, there is increasing evidence that epigenetic modifications are as important as genetic predisposition in the development of EH. Indeed, a complex and interactive genetic and environmental system exists to determine an individual’s risk of EH. Epigenetics refers to all heritable changes to the regulation of gene expression as well as chromatin remodelling, without involvement of nucleotide sequence changes. Epigenetic modification is recognized as an essential process in biology, but is now being investigated for its role in the development of specific pathologic conditions, including EH. Epigenetic research will provide insights into the pathogenesis of blood pressure regulation that cannot be explained by classic Mendelian inheritance. This review concentrates on epigenetic modifications to DNA structure, including the influence of non-coding RNAs on hypertension development.

## 1. Introduction

**Figure 1:**
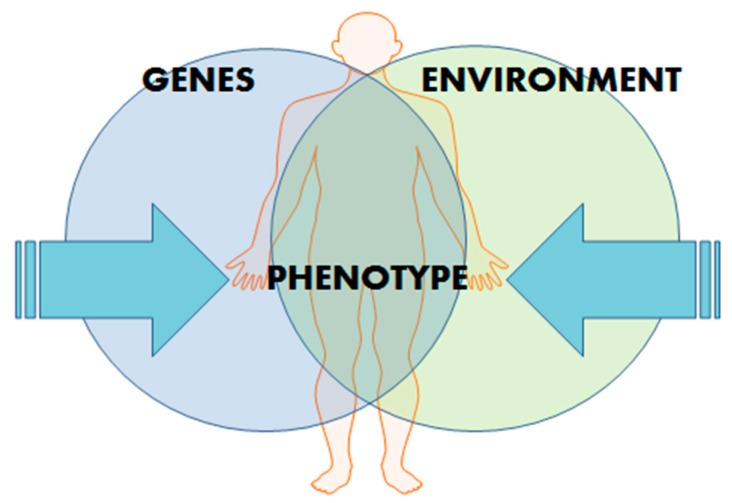
Influences on phenotype manifestation. Development of polygenic conditions, such as essential hypertension (EH), depend on a complex but interactive genetic and environmental system.

Hypertension (HT) affects more than 1 billion people globally and is a major risk factor for stroke, chronic kidney disease, and myocardial infarction [1]. Despite advances in our understanding of the pathophysiology of HT and the implementation of more effective treatment and prevention strategies, HT remains one of the world’s great public health problems [2]. The public health impact created by HT is due to its contribution to cardiovascular disease (CVD) events, such as heart attack, congestive heart failure, peripheral vascular disease, and stroke [3]. Essential hypertension (EH) is a complex, polygenic condition with no single causative agent. Furthermore, there is increasing evidence that epigenetic modifications are as important as genetic predisposition in the development of EH. Indeed, a complex and interactive genetic and environmental system exists to determine an individual’s risk of EH (Figure 1).

Epigenetic modification is recognised as essential in biological processes and in recent years there has been studies investigating its role in the development of specific pathologic conditions. Research into epigenetic mechanisms will provide insights into the pathogenesis of blood pressure regulation that cannot be explained by classic Mendelian inheritance. Previous reviews in this field report on the various epigenetic mechanisms impacting CVD [4,5,6] or they discuss a selection of epigenetic modifications in the context of HT but exclude some recent, important findings, such as the influence of long non-coding RNAs on HT [7,8,9,10]. This review concentrates on epigenetic modifications to DNA structure, including the influence of non-coding RNAs on EH development.

## 2. Epigenetics

Epigenetics refers to all heritable changes to the regulation of gene activity, without changes to the DNA sequence itself [11]. In other words, “epigenetics” refers to analysis of the human genome from the perspective of chromatin and chromosomes, as well as to the quintessential issue of the DNA sequence. Epigenetic modifications can be provoked by a variety of factors, including environmental influences during foetal and childhood development, chemical exposure, aging, dietary habits, and the use of recreational drugs and some prescription medications [10].

**Figure 2:**
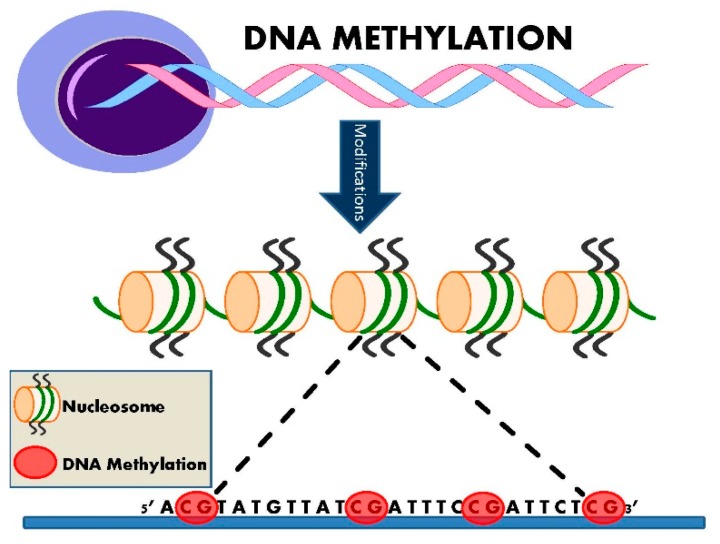
Epigenetic DNA methylation. DNA methylation involves the binding of a methyl group to the 5’ carbon of cytosine ring. This primarily occurs at CpG islands and results in inhibition of gene transcription, particularly if it occurs in the gene promoter region. It may also promote transcription if it is located at gene exons sites. DNA methylation is an essential biological process which is linked with several epigenetic phenomena including X-chromosome inactivation, genomic imprinting and repetitive element repression.

**Figure 3:**
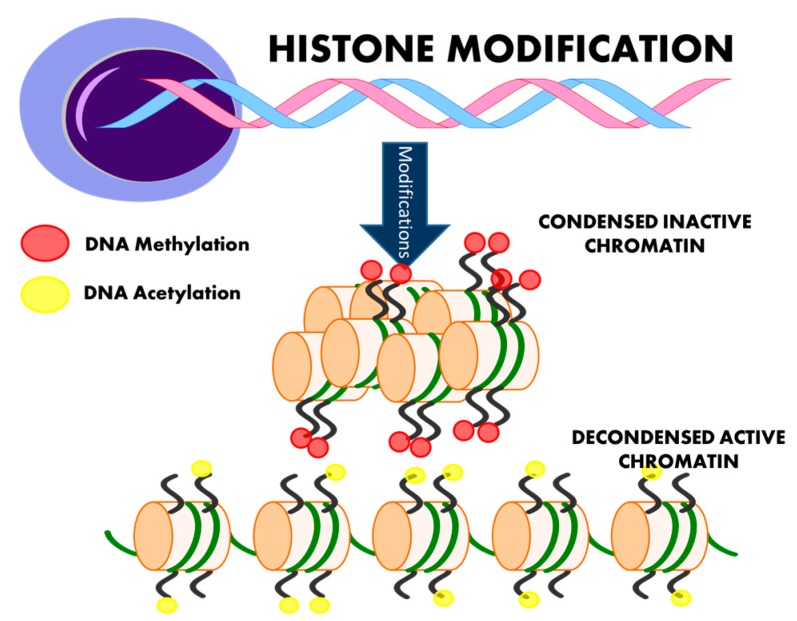
Histone modification. Post-translational changes in the form of histone modifications influence gene expression by controlling chromatin dynamics. Methylation is commonly associated with gene silencing and can directly interfere with the binding of transcription factors. Histone tail acetylation can increase the access of transcription factors to DNA by transforming condensed chromatin into a more relaxed structure.

**Figure 4:**
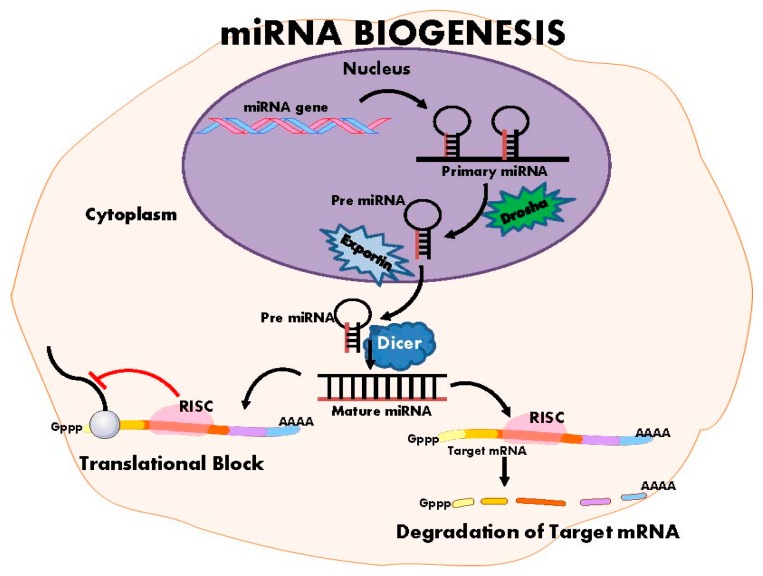
MicroRNA biogenesis. MiRNA biogenesis involves down-regulation of gene transcription via target mRNA degradation or by mRNA translation blockage. RISC: RNA-induced silencing complex.

The launch of international initiatives, the Human Epigenome Project [12], which first released data in 2003, and the International Human Epigenome Consortium [13] formed in 2010, attest to the rapid expansion of interest in epigenetics. Epigenetic inheritance is an essential mechanism that enables the stable propagation of gene activity states from one generation of cells to the next [14]; it is helping to resolve puzzling aspects of heredity, while explaining how the same genome can give rise to apparently contrasting phenotypic traits [15]. Chromatin consists of nucleosomes: DNA strands wrapped around histones. Several distinct biochemical processes can epigenetically modify both DNA and histones [10]. These include DNA methylation (Figure 2), post-translational histone modifications (Figure 3), and RNA-based mechanisms mediated by small non-coding microRNAs [16] (Figure 4).

## 3. DNA Methylation

Epigenetic DNA modification (methylation) occurs when a methyl group derived from *S*-adenosyl-l-methionine is bound to position 5 of the cytosine ring, forming 5-methyl-cytosine (5mC) [17] (Figure 2). This happens at specific dinucleotide sites called CpGs, as these are prone to such modifications [10]. CpG islands are short sequences of DNA with a high linear frequency of 5’-CpG-3’ sequences. The “P” denotes the phosphoesteric bond linking the cytosine and the guanine nucleotides [9]. CpG islands are often located in the promoter regions and approximately 40% of genes contain CpG islands in the end 5’ region (promoter, untranslated region, and exon1). The rest of the genome (intergenic and intronic regions) is considered CpG poor [17]. In healthy somatic cells, up to 90% of CpG dinucleotide sites are methylated, except for those located in the promoter region, which appear to be somewhat protected from such modification [17]. Non-CpG island DNA methylation has also been reported to influence protein-DNA interactions, gene expression and chromatin structure, and stability [18] (Table 1). Functionally, DNA methylation suppresses gene transcription, so DNA hypermethylation results in gene silencing [10].

Different degrees of DNA methylation have been correlated with variable onset, timing, and severity of EH (Table 1) [10,19]. Global genomic DNA methylation can be quantified by measuring the amount of 5-methyl cytosine present in a DNA sample. Smolarek *et al.* [19] found a correlation between decreased levels of 5mC in peripheral blood with an increase in EH grade severity. The concentration of 5mC was calculated as a ratio of spot intensities and a significant deficit in 5mC was observed in healthy controls, relative to levels found in the two severity grades of EH. The mean level of 5mC was 1.80 ± 0.69 in healthy controls and 1.14 ± 0.48 in all hypertensive subjects (specifically, 1.29 ± 0.50 grade 1, 0.99 ± 0.42 grade 2), indicating that global DNA methylation levels decrease as the severity of EH increases. However in this study, the percentage of 5mC was expressed as a coefficient “*R*” value, and the method for calculating “*R*” was not disclosed.

The most robust data on the involvement of methylation in blood pressure regulation comes from the study by Kato *et al.* [20]. Kato and colleagues performed a genome-wide association and replication study and identified genetic variants at 12 new loci that correlated with blood pressure modulation in 320,251 individuals of East Asian, European, and South Asian ancestry [20]. An investigation of the relationship between the sentinel blood pressure single nucleotide polymorphisms (SNPs) with local DNA methylation in 1904 South Asians (peripheral blood; Illumina Human Methylation 450 Bead Chip array (Erasmus Medical Centre, Rotterdam, The Netherlands)) revealed a two-fold enrichment between sequence variation and DNA methylation. Twenty-eight of the 35 sentinel SNPs were associated with one or more methylation markers [20], suggesting that DNA methylation may lie on the regulatory pathway linking sequence variation and blood pressure. Genes associated with the 12 newly identified SNP loci include those involved with vascular smooth muscle (*IGFBP3*, *KCNK3*, *PDE3A*, *PRDM6*) and renal function (*ARHGAP24*, *OSR1*, *SLC22A7*, *TBX2*) [20]. Investigation of cross-tissue patterns of DNA methylation at the leading 26 CpG sites associated with the sentinel SNPs showed that DNA methylation in the blood was closely correlated with methylation patterns of various tissues (liver, muscle, subcutaneous and visceral fat). While these potentially ground-breaking results indicate that methylation levels in blood may provide a proxy for methylation patterns in other tissues, further research is needed to investigate this correlation with a hypertensive affector organ, such as the kidney.

Initial DNA methylation research centred on correlating EH with the global level of 5mC [19], but more recent studies have focused on the methylation of specific DNA sequences. The hydroxysteroid dehydrogenase-11β2 enzyme (HSD11B2) converts cortisol to cortisone, which is found in circulatory concentrations up to 1000 times higher than aldosterone (the primary mineralocorticoid hormone and sodium (Na) transport modulator of the renin-angiotensin-aldosterone system (RAAS)) [10]. Cortisol and aldosterone bind mineralocorticoid receptors with equal affinity, but due to the difference in concentration of the hormones, cortisol has a larger role in sodium reabsorption by the kidneys and, in consequence, plays a larger role in fluid-related arterial pressure [10]. HSD11B2-mediated degradation of cortisol to cortisone is disrupted when the promoter region of the *HSD11B2* gene is hypermethylated [27,28]. The resulting imbalance in the active metabolites of cortisol and cortisone, tetrahydrocortisol (THF), and tetrahydocortisone (THE), respectively, promotes the onset of HT [27,28], in accord with the findings by Friso *et al.* [21]. Friso *et al.* [21] also found that *HSD11B2* promoter methylation was associated with EH, with parallel disruptions to the THF/THE ratio.

Due to the well-known involvement of the RAAS system on arterial pressure regulation, any changes in the activation status of RAAS-regulated genes have a pronounced effect on an individual’s potential to develop HT. This effect has been extensively tested in animal models of HT [29]. Hypomethylation of the promoter regions of the angiotensin II type 1b receptor gene in the adrenal glands of the maternal low protein (MLP) rat promoted HT through an exaggerated response to salt [29]. The same effect was seen in a second study, where maternal protein deficiency during pregnancy induced hypomethylation of the promoter regions of RAAS-responsive genes, such as angiotensin converting enzyme (ACE), causing predisposition to HT in offspring as well as cognitive deficits [22]. The human *PRCP* gene encodes a lysosomal prolylcarbopeptidase protein, which is implicated in cleavage of c-terminal amino acids linked to proline in peptides such as angiotensin II and III [30]. Methylation profiling conducted on young African males revealed that the *PRCP* gene was hypomethylated in EH subjects [23]. Similarly, when expression patterns of the angiotensin 1a receptor (*Atgr1a*) were analysed in both spontaneously hypertensive rat (SHR) and its normotensive control, the Wistar Kyoto rat (WKY), expression of *Atgr1a* was significantly increased by week 20 in the SHR. Bisulfite sequencing revealed that the *Atgr1a* promoter from endothelial cells in the aorta and mesenteric arteries of the SHR rats became progressively hypomethylated with age compared to their WKY counterparts [24], suggesting heightened *Atgr1a* expression in the SHR was related to the hypomethylation of the *Atgr1a* promoter and may have a role in the maintenance of high blood pressure. Somatic ACE (sACE) converts angiotensin I to the active form, angiotensin II and is, therefore, a key regulator of blood pressure [25]. Bisulfite sequencing conducted on cultured endothelial cells and Wistar Kyoto rats revealed that hypermethylation was associated with transcriptional repression of sACE, indicating possible epigenetic involvement of sACE modulation in HT [25].

Membrane transporters such as Na^+^-K^+^-2Cl^−^ cotransporter 1 (NKCC1), participate directly with fluid and electrolyte loss and therefore arterial pressure regulation [26]. NKCC1, expressed by the *Sic2a2* gene, mediates the transport of sodium, potassium and chloride into and out of the cells [10]. Changes in ion fluxes have been implicated in EH, particularly an augmented, passive influx Na^+^, K^+^, Rb^+^, and Cl^−^ in hypertensive vascular smooth muscle cells [26]. Hypomethylation of the *Sic2a2* gene promoter in the SHR aorta and heart, result in increased expression of NKCC1 and is positively correlated with HT [26].

Methyl CpG binding protein 2 methylates the norepinephrine transporter gene, silencing its expression [10]. Hypermethylation of the norepinephrine transporter gene, which transports norepinephrine and dopamine from the synaptic gap back to the pre-synaptic neuron, has been shown to lead to increased transport responsiveness, resulting in EH [31]. Furthermore, phenyl-ethanolamine *N*-methyltransferase (PNMT), which can act similarly to methyl CpG binding protein 2, has been shown to exacerbate the decrease in norepinephrine uptake, thereby enhancing the local and systemic catecholaminergic effect [31].

## 4. Histone Modification and Hypertension

DNA is packaged into the dynamic protein structure of chromatin, whose basic unit is the nucleosome. A nucleosome comprises of two copies each of the histone proteins H2A, H2B, H3, and H4 [32]. Post-translational modifications regulate gene expression by controlling the dynamics of chromatin. Modifications occurring at residues in histone tails include acetylation and methylation (Figure 3) as well as phosphorylation, sumoylation, and biotinylation [32].

Epigenetic histone modification occurs when the N-terminal tail is subjected to a variety of post-translational modifications [10]. Up to 60 possible modifications can occur. Specifically, the nucleotide lysine can be modified by methylation, acetylation, ubiquitylation, or sumoylation, whereas arginine can only be modified by methylation, and serine and threonine are modified only by phosphorylation [10]. While each histone modification pattern has a unique impact on the corresponding nucleosome, generally, the outcomes are similar: histone acetylation promotes gene transcription while deacetylation represses transcription; histone lysine methylation in position 79 inhibits while histone arginine methylation activates gene transcription; histone lysine hypermethylation or mono-methylation at position 9 can have opposing effects—respectively silencing or activating the target gene [9] (Table 2).

Histone modification affecting arterial pressure levels has been documented in a variety of human and animal tissues, including vascular smooth muscle. Vascular oxidative stress can contribute to endothelial dysfunction—a hallmark of HT—and the development of HT. A study by Bhatt *et al.* [39] documented the beneficial effects of resveratrol on endothelial function in the SHR. A later study of the mechanisms behind this effect was conducted by Han *et al.* [33]. Epigenetic modifications, in the form of up-regulated H3K27me3 expression was observed in the renal artery of a salt-sensitive hypertension model of the Wistar rat [33], possibly correlating with the improvement observed in HT status but this was not clear. Endothelial nitric oxide synthase (eNOS) is primarily responsible for the production of nitric oxide in the vascular endothelium, and plays a key role in the regulation of vascular tone [33]. eNOS activity is thought to be down-regulated in CVD. Fish *et al.* [34] found that eNOS expression appears to be controlled by cell-specific histone modifications. They also demonstrated that the nucleosomes of endothelial cells corresponding to the eNOS core promoter were enriched in lysine 9 of histone H3 and lysine 12 acetylation of histone H4, and of di- and tri-methylation of lysine 4 of histone H3. Fish *et al.* [34] observed that all of these epigenetic histone modifications were significantly important for the expression of eNOS [34].

The study by Riviere *et al.* [25] noting the modulation of sACE by CpG methylation also documented the involvement of histone deacetylases in the same process. Later findings of Lee *et al.* [35] further validated these observations. Riviere *et al.* [25] also found that tissues from SHRs exhibit higher expression levels of Ace1 mRNA and protein than those of WKY controls. They found the *Ace1* promoter regions of the SHR tissues were more enriched with H3Ac and H3K4me3, and concluded that *Ace1* is locally up-regulated in SHR tissues via histone code modifications [35].

In line with observations by Lee *et al.* [26], Cho *et al.* [36] found that levels of the membrane transporter *Nkcc1* mRNA and protein in the aortas of Sprague–Dawley (SD) rats were significantly increased after administration of an angiotensin II infusion. Cho *et al.* [36] found that acetylated histone H3 (an activating histone), was significantly increased together with greatly decreased trimethylated histone H3 (a deactivating histone) [36], suggesting that both histone modification and/or DNA demethylation have a role in the epigenetic up-regulation of *Nkcc1* during HT development.

Disruptor of telomeric silencing-1 (DOT 1), a methyl-transferase, enhances methylation of lysine 79 residue of histone H3 (H3K79) [10]. DOT1-mediated hypermethylation of H3 disrupts silencing of genes associated with maintaining telomere length during DNA repair, located in the telomeric regions of chromosomes [40]. This disruption correlates with a decrease in connective tissue growth factor transcription, increased intracellular cAMP and alterations in the adaption of blood vessels to stressors—associated with HT [40]. In Caucasians, DOT1-like histone H3K79 methyltransferase (DOT1L) is strongly associated with increased blood pressure in response to hydrochlorothiazide [37].

Activation of the renal sympathetic nervous system has long been thought to play a crucial role in the development of salt-reactive HT [41]. Overactivity of renal sympathetic pathways can promote the activation of several mechanisms which lead to increased sodium retention, including stimulation of renin release, reduced renal blood flow, and increased sodium reabsorption in the loop of Henle [42,43]. Serine-threonine kinases have a key role in renal tubular sodium reabsorption; specifically, the with-no-lysine (WNK) family of serine-threonine kinases [44]. In normotensive mice, a low-sodium diet decreases expression of renal WNK4, whereas a high sodium diet increases its expression [45,46]. Renal sympathetic activity can also promote sodium reabsorption via activation of the β-2 adrenergic receptor (β_2_AR) leading to cyclic AMP (cAMP) production, and increased activity of renal epithelium sodium channels (ENaC) [30]. WNK4 signalling targets ENaC, and may also mediate the prohypertensive effects of renal sympathetic overactivity. In addition to the classical cAMP pathway, cAMP modulates gene transcription via inhibition histone deacetylase-8 activity leading to increased histone acetylation [47,48]. Mu *et al.* [38] found that salt loading triggers β-adrenergic-mediated down-regulation of renal WNK4, increases salt retention, and consequently leads to the development of salt sensitive HT. WNK4 was recently found to be negatively regulated by the glucocorticoid receptor [49]. Mu *et al.* [38] also noted that the underlying mechanism down-regulating renal WNK4 is cAMP dependent modulation of histone acetylation, which consequently increased histone H3 and H4 acetylation. Acetylation of H3 and H4 seems to favour binding of the glucocorticoid receptor to a *WNK4* promoter region that contains a negative glucocorticoid-responsive element [38]. Mu *et al.* found WNK4 expression was inhibited via these pathways leading to overexpression of the sodium chloride co-transporter (NCC) and the onset of HT [38].

## 5. Non-Coding RNAs and Hypertension

Non-coding RNAs (ncRNA) are implicated in several epigenetic processes, notably small RNAs that can influence histone modifications and cytosine methylation which are connected with gene expression regulation [50]. NcRNA modification can produce effects analogous to classical DNA or histone epigenetic mechanisms described earlier, or can induce HT via separate, distinct processes. Essentially, epigenetics allow the same genome to show alternative phenotypes based on different epigenetic influences. Some of the most complicated and researched epigenetic phenomena including X-chromosome inactivation, parental imprinting and paramutation have an RNA contribution, either directly or indirectly [50]. While most ncRNAs have no identified function yet, it is conceivable that all are participants in epigenetic mechanisms which are yet to be clearly defined. NcRNAs have a strong influence on the “central dogma” of biology. The past view that all DNA was transcribed into RNA and then translated into protein has now been updated to include the fundamental role of ncRNAs on the regulation protein levels. Recently, several small and mid-sized ncRNAs have been described and are known to have a role in the regulation of transcription, post-transcription and translation. Small ncRNAs include PIWI-interacting RNAs (piRNAs), transcription initiation RNAs, and microRNAs (miRNAs) [8,51]. Mid-size ncRNAs include small nucleolar RNAs, promoter upstream transcripts, TSS-associated RNAs, and promoter-associated small RNAs. Long ncRNAs (lncRNAs) are most commonly associated with a reduction in transcription but may also have a role in the regulation of miRNA levels [8]. For a comprehensive review of miRNAs and blood pressure refer to the review paper by Marques *et al.* [51]. Here we will deliver a brief summary of some additional key miRNAs involved in HT as well as a review of lncRNAs.

MiRNAs are the most commonly studied small ncRNA; currently there is no research available investigating the involvement of the other types of small and mid-sized ncRNAs in EH. There are more than 1800 miRNAs in the genome, each approximately 22 nucleotides in length [52]. miRNAs down-regulate protein-coding genes by binding to target sequences in the 3’ (5’ targeting is uncommon but possible) untranslated region of a target mRNA, resulting in mRNA degradation or repression of mRNA translation [53] (Figure 4). Evidence suggests a single miRNA can regulate numerous mRNAs, so it is plausible that miRNAs are causative agents for complex deletions [53]. miRNAs are increasingly implicated as regulators of cardiovascular system [54], including modulation of arterial pressure [49] (Table 3).

In regards to the RAAS-regulated genes, it appears that miRNAs may also have a role modulating ACE mRNA transcription [29]. MiRNA has-miR-155 was found by Sethupathy *et al.* [55] to target the polymorphic sequence located in the 3’UTR of *AGTR1* mRNA. This site has a Mendelian C (minor) and an A (major) allele. The minor-C allele is associated with EH [55]. Has-miR-155 binds most effectively to the A allele, leading to a reduction in *AGTR1* mRNA in EH individuals who inherit this allele. Individuals with the C allele did not have reduced *AGTR1* mRNA and exhibited a greater pressor effect in response to angiotensin II [55]. Has-miR-155 is also found to be up-regulated in preeclampsia placentas; it regulates *AGTR1* expression in umbilical vein endothelial cells [56]. A recent genome-wide association study documented expression of mRNAs and miRNAs in kidneys from untreated EH individuals, which were removed as a consequence of renal cancer [57]. Two miRNAs (has-miR-181a and has-miR-663) with the ability to bind to the 3’UTR of renin mRNA were found to be under-expressed in EH. These miRNAs were able to regulate the expression of a reporter gene and renin-mRNA itself, which explains over-expression of renin mRNA seen in EH kidney [57]. In reference to DOT1 causing hypermethylation of H3 [37] (mentioned earlier), small interfering RNAs (siRNA) have been shown to have an opposite effect by silencing the expression of the *DOT1* gene [61]. Furthermore, siRNAs have been observed to have a role in modulating the RAAS system [61]. Angiotensin II was shown to induce a contractile response in smooth muscle cells extracted from mice overexpressing p22phox, a subunit of NADPH oxidase responsible for activating NADPH oxidase and generating superoxide anions in atherosclerotic plaques in human blood vessels [62]. siRNA targeting p22phox inhibits the contractile response from angiotensin II, lowering blood pressure [58].

Long non-coding RNAs (lncRNAs), a heterogeneous group of non-coding transcripts longer than 200 nucleotides, regulate their targets by influencing epigenetic control, mRNA processing, translation or chromatin structure. LncRNAs have been implicated in several biological processes, including transcriptional regulation by epigenetic mechanisms [63]. An exemplary representation is X-chromosome inactivation present in the female genome of mammals which involves the inactivation of one copy of a female sexual chromosome by DNA methylation. This process is coordinated by the X-inactive specific transcript (XIST) and the XIST antisense transcript (TSIX) ncRNAs, very long ncRNAs whose expression can be regulated by epigenetic mechanisms such as DNA methylation [50].

Large intergenic non-coding RNAs (lincRNAs) have been identified both as modulators of health development [64,65,66] and disease states [67], and may regulate the expression of neighbouring genes and distant loci [63]. LincRNAs are more tissue specific than are protein-coding genes [59], suggesting they have selective functions in different tissues. Cabili *et al.* [59] have described the annotation and tissue-specific expression of all lincRNAs. Their gene ontology analyses were based on lincRNA expression in different tissues [59]. The protein-coding genes located close to lincRNA loci included genes previously associated with EH and blood pressure regulation, for example, those encoding corticotropin-releasing hormone [59], suggesting many lincRNAs have an enhancer-like function that promotes transcription of their neighbouring coding genes [68]. LncRNAs have a potential to influence CVD, stroke, blood pressure and HT. Seven blood pressure candidate genes *ADD3*, *NPPA*, *ATP1A1*, *NPR2*, *CYP17A1*, *ACSM3*, and *SLC14A2* were connected with *cis*-lncRNA transcripts [60]. Of these, the lncRNA NPPA-AS was selected for further investigation which revealed NPPA-AS has a demonstrated influence on the splicing of the natriuretic peptide precursor A (*NPPA*) gene (present in cardiac hypertrophy and heart failure) and, therefore, has potential CVD involvement [60].

## 6. Conclusions

**Figure 5:**
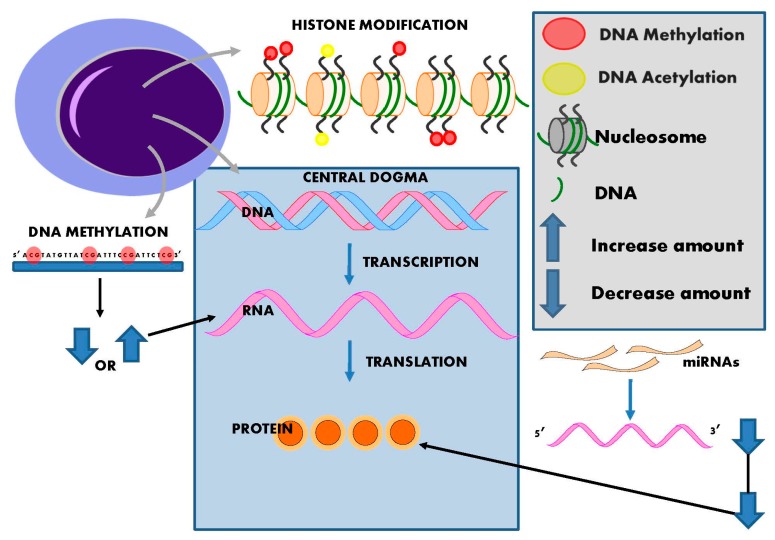
Impact of epigenetic modifications. DNA wrapped around nucleosomes are made up of four pairs of histone proteins. Histone proteins are prone to epigenetic modification, primarily acetylation and methylation but also phosphorylation, sumolyation, and biotinylation. These modifications change the formation of chromatin to either an open (active) or closed (inactive) state, thereby altering their transcriptional activity. DNA methylation changes the structure of DNA itself, allowing active transcription or silencing of genes. The outcome of DNA methylation is dependent on the location of the methylated site. miRNAs primarily target the 3’ UTR of mRNA (although 5’ targeting is possible). This negatively regulates the quantity of the encoded protein produced via degradation of mRNA molecules or by post-transcriptional regulation of mRNA stability.

We are entering a new era of understanding how the genome interacts with the environment to affect disease pathogenesis. There is now emerging evidence that epigenetic, as well as genetic, factors are key players in regulating and maintaining blood pressure, and strong evidence for a complex interaction of genetic and environmental factors that influence the risk of HT in each individual (Figure 5). Many epigenetic studies are, however, limited by the fact that only blood is studied rather than the effector tissues. The utility of blood methylation status in epigenetic research is yet to be determined. Furthermore, the polygenic complexity of HT and the limited knowledge on some of the non-coding RNAs makes it more challenging to decipher the exact mechanisms involved. Further studies in humans and in animal models will be needed to elucidate the exact mechanisms involved and to determine possible therapeutic applications.

## Figures and Tables

**Table 1:** Summary of methylation findings in relation to hypertension.

Reference	Tissue Type/Sample Size	Findings
Smolarek, *et al.* [19]	Human: Peripheral blood; 60 with EH; 30 controls	Mean 5mC amount in DNA significantly decreased as HT severity increased.
Kato, *et al.* [20]	Human: Peripheral blood, cord blood, muscle, liver, fat; 320,251 individuals of East Asian, European and South Asian ancestry	Multiple genetic variants involved with vascular smooth muscle (*IGFBP3*, *KCNK3*, *PDE3A*, *PRDM6*) and renal function (*ARHGAP24*, *OSR1*, *SLC22A7*, *TBX2*) discovered to be correlated with BP modulation. Two-fold enrichment discovered between DNA methylation and sentinel blood pressure SNPs, providing evidence for DNA methylation role in blood pressure regulation.
Friso, *et al.* [21]	Human: Peripheral blood; 25 with EH; 32 with prednisone therapy	*HSD11B2* gene promoter methylation associated with EH onset via disruption to THF/The ratio.
Goyal, *et al.* [22]	Rat: Tissues: brain; 20 MLP pups, 17 control pups	Hypomethylation of RAAS system genes such as ACE resulting in HT in offspring.
Wang, *et al.* [23]	Human: Peripheral blood; 8 EH; 8 control	*PRCP* gene hypomethylated in EH, linked to disruption in cleavage of angiotensin II and III.
Pei, *et al.* [24]	Rat: Tissue: aorta; 6 Spontaneously HT; 6 WKY control	*Atgr1a* gene progressively hypomethylated as SHR age progressed. Indicating increased expression of *Atgr1a* in aging SHR.
Riviere, *et al.* [25]	Rat: Cultured endothelial cells from WKY	Hypermethylation associated with trasciptional repression of sACE, indicating a role for epigenetics in sACE modulation during HT.
Lee, *et al.* [26]	Rat: Tissues: aorta, heart; SHR and WKY	Hypomethylation of *Sic2a2* gene lead to increased expression of NKCC1 which was positively correlated with HT.

EH: essential hypertension; MLP: maternal low protein; HT: Hypertension; SHR: spontaneously hypertensive rat, WKY: Wistar Kyoto rat; 5mC: 5-methyl-cytosine; ACE: angiotensin converting enzyme; RAAS: renin-angiotensin-aldosterone system; sACE: somatic ACE; SNPs: single nucleotide polymorphisms; NKCC1: Na^+^-K^+^-2Cl^−^ cotransporter 1; BP: blood pressure.

**Table 2:** Summary of histone modification findings in relation to hypertension.

Reference	Tissue Type/Sample Size	Findings
Han, *et al.* [33]	Rat: Tissue: Aorta, renal artery; SHR and WKY	Up-regulated histone modifier H3K27me3 in renal artery of SHR correlated with HT improvement after resveratrol intake.
Fish, *et al.* [34]	Human: Umbilical vein endothelial cells	Endothelial cell nucleosomes corresponding to eNOS enriched in various histones relevant to eNOS expression.
Lee, *et al.* [35]	Rat: Tissue: adrenal gland, aorta, heart, kidney, liver, and lung. SHR and WKY	Higher expression of *Ace1* mRNA & protein in SHR. *Ace1* promoter enriched with H3Ac and H3K4me3 in SHR.
Cho, *et al.* [36]	Rat: Tissue: Mesenteric artery, aorta; SD and Sham rat.	*Nkcc1* up-regulated in SD rat. Acetylated histone H3 up-regulated, trimethylated histone H3 down-regulated.
Duarte, *et al.* [37]	Human: Peripheral blood; First sample: 206 mixed sex, normotensive; Second sample: 730 mixed sex, HT and normotensive.	DOT1L strongly associated with increased BP in Caucasians. Possibly via mediation of hypermethylation of H3.
Mu, *et al.* [38]	Mouse: Tissue: Kidney; norepinephrine infused-C57 BL/6j, *Adrb1* knockout and *Adrb2* knockout mice.	WNK4 down-regulation caused increased H3 & H4 acetylation, leading to overexpression of NCC and therefore promoting HT onset.

eNOS: endothelial nitric oxide synthase; SD: Sprague–Dawley rat; DOT1L: DOT1-like histone H3K79 methyltransferase; NCC: sodium chloride co-transporter; WNK4: with-no-lysine kinase 4.

**Table 3:** Summary of non-coding RNAs in relation to hypertension.

Reference	Tissue Type/Sample Size	Findings
Goyal, *et al.* [22]	Rat: Tissues: brain; 20 maternal low protein pups, 17 control pups	mmu-miR-27a and mmu-miR-27b regulate *ACE1* and was upregulated (3.3- and 8.8-fold respectively) in MLP rat; mmu-mir-330 regulates angiotensin II type 2 receptor (A$T_{2}$) and was downregulated 3.5-fold in MLP rat.
Sethupathy, *et al.* [55]	Human: Fibroblasts from monozygotic twin; *n* = 2	Has-miR-155 binds to 3’UTR of AGR1 mRNA “A” allele causing a reduction in *AGTR1* mRNA, reducing the pressor effect in response to angiotensin II.
Cheng, *et al.* [56]	Human: Endothelial cells from pre-eclamptic placentas	Has-miR-155 up-regulated in preeclampsia placentas, indicating involvement in regulation of *AGTR1*.
Marques, *et al.* [57]	Human: Tissue: kidney; Sample 1: 42 mixed sex, Polish individuals of mixed HT status; Sample 2: 22 male only, mixed HT status. All samples untreated for HT	Has-miR-181a & has-miR-663 is able to bind 3’UTR of renin mRNA, found to be underexpressed in EH. These miRNA able to regulate renin mRNA directly, explaining overexpression of renin in EH kidney.
Wang, *et al.* [58]	Mouse: Tissue: Mesenteric arterioles; 16 male C57Bl/5 mice; 16 sham mouse control	siRNA targeting p22phox mRNA demonstrated inhibition of contractile response from angiotensin II, consequently lowering BP.
Cabili, *et al.* [59]	Human: Tissue: 24 various and cells lines; 24 human samples	lincRNAs may promote the transcription of their neighbouring coding genes, including those implicated in EH and BP regulation.
Annilo, *et al.* [60]	Human and Mouse: Various tissues and cell lines; n = not disclosed	Seven blood pressure candidate genes *ADD3*, *NPPA*, *ATP1A1*, *NPR2*, *CYP17A1*, *ACSM3* and *SLC14A2* were connected with *cis*-lncRNA transcripts.
